# Hepatitis B vaccination coverage and the determinants of vaccination among health care workers in selected health facilities in Lusaka district, Zambia: an exploratory study

**DOI:** 10.1186/s40557-017-0191-y

**Published:** 2017-08-10

**Authors:** Namwaka Mungandi, Mpundu Makasa, Patrick Musonda

**Affiliations:** 1 Ministry of Health, Nchanga North General Hospital, P.O Box 10063, Chingola, Zambia; 2 School of Public Health, University of Zambia, P.O Box 50110, Lusaka, Zambia

**Keywords:** Hepatitis B, Healthcare workers, Sharp injuries, Vaccinations

## Abstract

**Background:**

Hepatitis B is a viral infection of the liver and causes both acute and chronic disease. It is transmitted through contact with an infected person’s bodily fluids. It is an occupational hazard for healthcare workers and can be prevented by the administration of a vaccine. It is recommended that healthcare workers be vaccinated against vaccine preventable diseases including hepatitis B. The study objective was to determine the prevalence and determinants of hepatitis B vaccination among healthcare workers in selected health facilities in Lusaka.

**Methods:**

The study took place in seven health facilities across Lusaka district in Zambia. A total sample size of 331 healthcare workers was selected of which; 90 were nurses, 88 were doctors, 86 were laboratory personnel and 67 were general workers. A self-administered structured questionnaire was given to a total of 331 healthcare workers. Investigator led stepwise approach was used to select the best predictor variables in a multiple logistic regression model and all analyses were performed using STATA software, version 12.1 SE (Stata Corporation, College Station, TX, USA).

**Results:**

Only 64(19.3%) of the healthcare workers were vaccinated against hepatitis B, with 35 (54.7%) of these being fully vaccinated and 29 (45.3%) partially vaccinated. Analysis showed that; age of the healthcare worker, sharp injuries per year and training in infection control were the variables that were statistically significant in predicting a healthcare worker’s vaccination status.

**Conclusion:**

It is reassuring to learn that healthcare workers have knowledge regarding hepatitis B and the vaccine and are willing to be vaccinated against it. Health institutions should bear the cost for vaccinating staff and efforts should be made for appropriate health education regarding hepatitis B infection and its prevention. Establishment of policies on compulsory hepatitis B vaccination for healthcare workers in Zambia is recommended.

## Background

Health care workers (HCWs) are defined as persons whose activities involve contact with patients, blood or other body fluids in a health care, laboratory, or public health setting; for example employees, attending clinicians, public safety workers, students, contractors or volunteers [1]. Due to the nature of work, HCWs are at greater risk of infections from patients. Blood borne pathogens particularly, hepatitis B virus (HBV), hepatitis C virus (HCV) and human immunodeficiency virus (HIV) have been known to infect HCWs [2]. It is estimated that more than two billion people are infected with HBV worldwide and about 350 million of them suffer from chronic hepatitis B infection mainly liver cirrhosis and hepatocellular carcinoma [3]. Hepatitis B is endemic in almost all parts of the world and 60% of the world population lives in endemic areas [4]. It is also estimated that about 500–600 HCWs are hospitalized annually due to exposure to blood products [5]. The Center of Disease Control (2014) reports that the prevalence of hepatitis B chronic infection is particularly high in sub Saharan Africa ranging from 7 to 26%. HCWs are at a high risk of hepatitis B due to occupational exposure to blood, and incidence of infection among them has been estimated to be 2 to 4 times higher than in the general population [5]. Hepatitis B causes acute infection and chronic infection [6], and can cause scarring of the liver, liver failure, liver cancer, and even death [7]. Infection can be acquired through contact with infected blood and other bodily fluids such as semen, vaginal secretions, and open sores. The virus remains infectious for prolonged periods on environmental surfaces and is transmissible in the absence of visible blood [6]. HBV has long been recognized as an occupational hazard for HCWs, including HCW trainees [6]. As part of occupational safety measures, the World Health Organization states that all HCWs are required to be vaccinated against HBV. Unfortunately, the World Health Organization also estimates that HBV vaccination coverage amongst HCWs is only 18–39% in low and middle income countries and 67–79% in high income countries. HCWs have an increased risk of acquiring hepatitis B as they attend to infected patients and thus are exposed to blood and body secretions of patients [4]. The risk of acquiring hepatitis B infection from an HBV carrier ranges from 6 to 30% [8]. The risk of transmission of an infection from HCWs to patients is also great. There has been an increase in the number of such reports. Since 1972, 50 outbreaks have been reported in which a total of 48 infected HCWs, (39 surgeons) transmitted the infection to approximately 500 people [9]. Highly viremic HBV carriers with Hepatitis B antigen transmit the virus on average to 4% of their patients [9]. Nurses report a higher number of significant exposures than any other group. Most (58%) report exposure during a procedure and some (37%) after the procedure [8]. Occupational hazards in health facilities in Zambia are high with sharps injury rate per worker at 1.3 injuries per annum [10]. Despite the fact that infection is preventable through vaccination and post-prophylactic exposure, HCWs are unaware of the risks of HBV infection and appropriate preventative measures [11]. It is estimated that the prevalence of HBV in Zambia is at 12% in the general population which is amongst the highest in the world [3]. A study conducted in 5 health facilities in Zambia shows that the prevalence of hepatitis B vaccination amongst HCWs is only 8% [10]. As far as we are aware, this study is the only published study that investigates hepatitis B vaccination among health workers in Zambia. The study objective was to determine the proportion of health care workers vaccinated against hepatitis B and the determinants of vaccination among HCWs.

## Participants and methods

This was an exploratory study that was conducted in seven (7) health facilities across Lusaka district in Zambia from October 2015 to March 2016. These health facilities were level I, level II and level III health facilities. Nurses, doctors, laboratory technologists/scientists and general workers who had worked in the health facility for at least six months and had consented to take part in the study were enrolled. A stratified sampling technique was used to select the participants. The strata were the nursing department, surgery department, laboratory department and maintenance department. The selection of participants from each stratum was done through simple random sampling technique using a table of random numbers. A total number of 331 health care workers were selected from these health facilities, which included 90 nurses, 88 doctors, 86 laboratory technologists/technicians and 67 general workers.

Data was collected using a semi-structured questionnaire that was self-administered. The questionnaire comprised of six categories namely; demographic data, occupational data, occupational hazards, history of training in infection control, knowledge of recommended HBV vaccination coverage for HCWs and HBV vaccination information. The dependent variable was hepatitis B vaccination status which had a binary outcome (yes or no), and the independent variables included; age (years), gender (male or female), work experience (years), sharp injuries/year (number of sharp injuries), sector (public or private), training in infection control (yes or no), facility level (level I, II or III) and knowledge of hepatitis B (poor, fair, good or excellent). The collected data was then coded and entered into STATA version 12.1. Descriptive statistics such as the mean, standard deviations, numbers and percentages were performed and investigator led stepwise approach was used to select the best predictor variables in a multiple logistic regression model.

## Results

The total sample size consisted of 331 health care workers with a mean age of 35 years old (standard deviation 0.5). There were 153 (46.2%) males and 178 (53.8%) females in the study population. In the total sample, 90 (27.2%) were nurses, 86 (26.0%) where laboratory personnel, 88 (26.6%) were doctors and 67 (20.2%) general workers. The sample size was evenly distributed among the professionals. The mean work experience of the study population was 5.8 years, with the shortest being 1 year and longest 32 years. The average number of sharp injuries per health worker per year was 0.3 (SD 0.04). A majority of health care workers had excellent knowledge in hepatitis B (47.7%), and these were doctors, nurses and laboratory personnel. Those that had poor knowledge (13.9%) were mostly general workers. A total number of 141 (42.9%) health care workers were not trained in infection control at the health facilities they work for, whereas 190 (57.4%) health care workers were trained in infection control. From the total number of participants, 78 (23.6%) were from a Level I health facility, 92 (27.8%) from a level II health facility and 161 (48.6%) from a level III health facility. From these, 270 (81.75%) were from the public sector and 61 (18.43%) were from the private sector. The total number of healthcare workers vaccinated against hepatitis B was 64 (19.3%) and those not vaccinated against hepatitis B was 267 (80.7%). Table 1 shows the descriptive statistics of the study population.

**Table 1:** Descriptive Statistics of Study Population

Variable	Vaccinated		*P*-value
	No	Yes	
	*n* = 267	*n* = 64	
Age (mean, SD)	34.5(0.5)	37.3(1.2)	*p*< 0.0001^a^
Sex (frequency, percentage)			
Male	121 (79.1)	32 (20.9)	
Female	146 (82.0)	32 (18.0)	0.500^b^
Profession (frequency, percentage)			
Nurses	76 (84.4)	14 (15.6)	
Laboratory personnel	58 (67.4)	28 (32.6)	*p*< 0.0001^b^
Doctors	70 (79.5)	18 (20.5)	
General workers	63 (94.0)	4 (6.0)	
Work Experience (mean, SD)	5.6 (0.3)	6.2 (0.5)	*p*< 0.0001^a^
Sharps injuries/year (median, range)	0 (0–5)	0(0–5)	0.028^a^
Knowledge on Hepatitis B			
Poor	41(89.1)	5(10.9)	
Fair	22 (84.6)	4(15.4)	
Good	81 (80.2)	20(19.8)	0.363^b^
Excellent	123 (77.9)	35(22.1)	
Sector			
Public	218 (80.7)	52 (19.3)	0.941
Private	49 (80.4)	12 (19.6)	
Facility Level (frequency, percentage)			0.014^b^
Level I	112 (82.3)	24 (17.7)	
Level II	33 (97.1)	1 (2.9)	
Level III	122 (75.8)	39 (24.2)	
Training in infection control			
No	123 (87.2)	18 (12.8)	
Yes	144 (75.8)	46 (24.2)	0.009^b^

^a^Two-sample test with unequal variance ^b^Chi-square test

Table 2 shows unadjusted and adjusted analysis of the independent variables to vaccination status. From this table the variables that initially were associated with hepatitis B vaccination status were age (*p* = 0.01), profession (*p* = 0.002), sharp injuries/ year (*p* = 0.02) and facility level (*p* = 0.019). But after an investigator led-stepwise approach, which eliminates the least significant variables in a multiple logistic regression model, the variables: age (*p* = 0.042), sharp injuries per year (*p* = 0.008) and training in infection control (*p* = 0.018) were the best predictors for whether a health care worker was vaccinated against hepatitis B or not. Table 3 shows the exact odds ratios after the final model was achieved.

**Table 2:** Unadjusted and Adjusted analysis of Independent variables to vaccination status

Independent variable	Unadjusted Odds ratio (CI)	*P*-value	Adjusted Odds ratio (CI)	*P*-value
Age	1.03 (1.00–1.1)	0.28	1.05 (1.0–1.1)	0.01*
Sex				
Male	1.00		1.00	
Female	0.8(0.4–1.4)	0.50	0.9 (0.5–1.6)	0.60
Profession				
Nurse	1.00		1.00	
Lab personnel	2.6 (1.3–5.4)	0.009	4.1 (1.6–10.2)	0.002
Doctor	1.4 (0.6–3.01)	0.396	1.6 (0.6–4.0)	0.328
General worker	0.3 (0.1–1.1)	0.072	0.2(0.03–1.5)	0.115
Work Experience	1.02 (0.9–1.1)	0.93	0.9 (0.9–1.03)	0.252
Sharps injuries/year	1.6(1.2–2.1)	0.004	1.5 (1.1–2.0)	0.020
Knowledge of hepatitis B				
Poor	1.00		1.00	
Fair	1.5 (0.4–6.1)	0.580	0.6 (0.07–4.6)	0.613
Good	2.0 (0.7–5.8)	0.188	0.4 (0.06–3.3)	0.44
Excellent	2.3 (0.9–6.3)	0.097	0.03 (0.04–2.2)	0.233
Sector				
Public	1.00		1.00	
Private	1.02 (0.5–2.1)	0.07	1.6(0.5–2.7)	0.723
Facility Level				
Level I	1.00		1.00	
Level II	0.1(0.01–1.1)	0.06	0.7 (0.07–0.6)	0.019
Level III	1.5(0.8–2.6)	0.17	1.8 (0.8–4.0)	0.122
Training in infection				
No	1.00		1.00	
Yes	2.2 (1.2–4.0)	0.01	1.9 (0.9–3.8)	0.071

*Variables chosen to be statistically significant when *p* < 0.05

**Table 3:** The best fit model of variables that predict hepatitis B vaccination status in health care workers

Variable	Adjusted odds ratio(CI)	*P*-values
Age	1.03 (1.0–1.06)	0.042
Sharp injuries per year	1.5(1.1–2.1)	0.008
Training in infection control		
No	1.0	
Yes	2.1 (1.1–3.2)	0.018

The interpretation of the values in Table 3 are as follows; With every one-year increase in age of the healthcare worker, they are 1.03 times more likely (95% CI 1.0–1.06 *p* = 0.042) of being vaccinated against hepatitis B adjusting for sharp injuries per year and training in infection control of the health care worker. With every one increase in sharp injuries per year experienced by a health care worker, they are 1.5 times more likely (95% CI 1.1–2.06, *p* = 0.008) to be vaccinated against hepatitis B, adjusting for age of health care worker and an individual’s training in infection control. Health care workers who were trained in infection control were 2.1 times more likely (95% CI 1.1–3.2, *p* = 0.018) to be vaccinated against hepatitis B compared to those that were not trained in infection control, adjusting for the age of the health care worker and sharp injuries per year experienced by the health care worker.

## Discussion

The findings of this research suggest that there is indeed a low number (64/331, 19%) of HCWs vaccinated against hepatitis B in Lusaka district. In this study 281 (85.0%) of the HCWs knew that a vaccine against hepatitis B exists and 321 (97.0%) of the HCWs were willing to get vaccinated if the vaccine were to become available in their institution. A high knowledge base on the existence of the vaccine among HCWs and high willingness to be vaccinated suggests that the reason for low vaccination prevalence among HCWs is not by their own doing. Perhaps low vaccination rates can be due to the unavailability of the vaccine in the institution due to a non-stringent policy on the vaccination of HCWs.

Other studies have shown some interesting findings, for example, a study done in Uganda, found the prevalence of hepatitis B vaccination among HCWs at 38.4% [12] and 83.6% in Kuala lumpa [13]. In this study, out of the 64 HCWs that were vaccinated against hepatitis B, 35 (54.7%) had completed full vaccination (a total of 3 doses) and 29 (45.3%) had received either one or two doses. The study done in Uganda showed that only 6.2% of the HCWs had completed full vaccination which falls short to the 54.7% reported in this study. In Burkina Faso, 47.7% of HCWs had received at least one dose of hepatitis B vaccine and only 10.9% had received full vaccination [14]. South Africa also had a higher proportion of HCWs vaccinated against hepatitis B at 67.9%, however only 19.9% of these had received full vaccination [15]. In contrast to these observed low rates of full vaccination coverage in Africa, higher rates of 75.0% and 93.0% were reported among HCWs in the United States [16] and France [17] respectively.

Even though profession of the HCW was not significantly associated with vaccination status, it has been shown to be a strong predictor for hepatitis B vaccination. Literature shows that more doctors are vaccinated against hepatitis B compared to other HCWs [18]. This was not the case in this study with only 18 (5.4%) doctors being vaccinated against hepatitis B compared to laboratory personnel that had 28 (8.5%) HCWs being vaccinated. Most hospital laboratories in Zambia are taking part in enhancing the quality of work in their laboratories and one of the requirements to attain international accreditation is to have the laboratory personnel vaccinated against vaccine preventable diseases which include hepatitis B. This might be one of the reasons why the laboratory personnel had more vaccinated HCWs compared to other groups. The doctors that were vaccinated in this study consisted mainly of recent graduates and doctors that studied abroad. In Zambia, medical students are offered the hepatitis B vaccination under their regulatory body (Zambia Medical Association) at a fee. This could explain why the recently graduated doctors were vaccinated. Only 14 nurses (94.2%) were vaccinated against hepatitis B and they consisted of the largest group sampled (90 nurses). Most nurses vaccinated against hepatitis B was as a result of post prophylactic procedures mainly due to sharp injuries. The least vaccinated HCWs were the general workers, with only 4 (1.2%) being vaccinated against hepatitis B.

In this study, HCWs that had experienced at least one sharp injury in a year were 51 out of the total of 331 health care workers (15.4%). A study done in Pakistan reported that health care workers having experienced at least one sharp injury in a year was at 44% [19] with the highest frequency being in doctors. In this study nurses (37.3%) experienced the most sharp injuries per year at an average of 2 sharp injuries per year, followed by doctors (29.4%) at 1.6 sharp injuries per year, laboratory personnel (23.5%) at 1.2 sharp injuries per year and general workers (5.8%) at 1 sharp injury per year. In this study it is reported that the more sharp injuries per year a health worker experiences the more likely they are to be vaccinated against hepatitis B. Although the percentage of sharp injuries in this study is not too high, it should be minimized as it is one of the most significant modes of transmission. Moreover, needle prick injuries pose a greater risk than splashes and those from hollow-bore needles. Sharp injuries can be prevented by always wearing gloves, properly discarding needles and minimizing the contact with blood products of infected patients. HCWs that experienced a sharp injury would undergo post-prophylactic procedures (PEP), with a majority of them undergoing HIV PEP. The remainder of the health workers either did not undergo PEP or they underwent hepatitis B PEP which consists of taking 3 doses of the vaccine making them immunized against hepatitis.

The age of the health worker was also a determinant for hepatitis B vaccination. According to the analysis, with every increase in age a health care worker is 1.03 times more likely to be vaccinated against hepatitis B adjusting for sharp injuries per year and training in infection control. The odds ratio found in this study is not particularly high, but it does support literature findings that highlight that age indeed is a determinant for hepatitis B vaccination in HCWs. Ogoina states that, “it is plausible that younger health care workers had poorer vaccine uptake due to their lower access to hepatitis B vaccine or poorer knowledge of the need for hepatitis B vaccination” [18]. Due to more experience, older HCWs are more knowledgeable about hepatitis B and its risks and health impacts and are more likely to take extra precaution to prevent themselves from contracting hepatitis B. This would suggest that health education in safety practices be strengthened in colleges/universities in order for young graduates looking for employment in clinical setting be well informed about their options in preventing themselves from infection with hepatitis B.

All HCWs must be trained in ways to protect themselves from acquiring diseases in their work environment. Truth be told, HCWs are exposed to many diseases each and every day due to the contact that they have with patients and patients’ bodily fluids. All health institutions must make sure that their staff are trained in infection control practices in order to effectively protect themselves from acquiring diseases from the hospital environment in which they work. This study has shown that a health care worker trained in infection control is more likely to be vaccinated against hepatitis B compared to a health care worker not trained in infection control. Prior training in infection control of a HCW means they are more likely to be vaccinated against hepatitis B [18]. It is also suggested that training of a HCW in the practice of standard precautions makes them more likely to be vaccinated against hepatitis B [19].

### Limitations of the study

The sample size of 331 was small for the study because of limited resources, and research on a similar study must be conducted to include a larger sample size. Information on whether a HCW knew their hepatitis B status and hepatitis B antibody titre was not collected. This would have helped in accessing the relevancy of a HCW being vaccinated against hepatitis B. True vaccination status of the HCWs is questionable because vaccination certificates were not being produced. However, the results give an idea of the prevalence of hepatitis B vaccination among HCWs.

## Conclusion

The study identified a low coverage of hepatitis B vaccination among health care workers. The elimination of the transmission of hepatitis B to HCWs through the contact of infected patients’ bodily fluids and vice versa can be achievable through the vaccination of HCWs against hepatitis B. From this study it has been shown that the age, training in infection control and sharp injuries per year are the best predictors of hepatitis B vaccination in HCWs.

### Recommendations

Further studies must be done to improve the generalizability of these results to HCWs in Lusaka district. Efforts should be made to increase hepatitis B vaccination coverage among all HCWs, especially those at greatest risk for exposure to blood or other potentially infectious material. Hospitals also need to identify successful vaccination strategies focused on exposed, but unvaccinated HCWs. In order to increase the number of HCWs vaccinated against hepatitis B, health institutions should bear the cost for vaccinating their staff. The Ministry of Health Zambia must also implement and strengthen its policy on hepatitis B vaccination among HCWs, making it compulsory for HCWs to be vaccinated against hepatitis B in Zambia by the inclusion of hepatitis B education during orientation programs for new interns or new staff.

## Abbreviations

HBV: Hepatitis B virus
HCW: Health care worker
